# Tell me where it is: Selective difficulties in spatial language on the autism spectrum

**DOI:** 10.1177/1362361320921040

**Published:** 2020-06-04

**Authors:** Agata Bochynska, Kenny R. Coventry, Valentin Vulchanov, Mila Vulchanova

**Affiliations:** 1 Norwegian University of Science and Technology (NTNU), Norway; 2 New York University, USA; 3 University of East Anglia, UK

**Keywords:** developmental delay, linguistic development, selective deficits, spatial language, spatial prepositions

## Abstract

Proficient use of spatial terms such as *under, to the left of* or *in front of* is a central component of daily communication and is important in the development of language and spatial cognition. Here we examine spatial language abilities in intellectually high-functioning individuals with autism spectrum disorder, an area previously overlooked in autism research. Twenty-five high-functioning individuals with ASD and 25 typically developing controls, matched for chronological age and cognitive abilities, completed a novel battery tapping a broad range of spatial language abilities. We report selective difficulties in the production of spatial terms and spatial description recall in high-functioning ASD. Overall verbal abilities did not account for the observed group differences. Crucially, however, the intensity of autism spectrum traits predicted individual performance in both spatial language production and spatial description recall. We discuss the theoretical implications of these findings and explore their significance for both clinical practice and intervention.

**Lay abstract:**

How we think and talk about space is an essential ability, necessary for understanding the world around us. We recruit spatial thinking every day when finding our way or using tools but also in more advanced tasks, such as reading complex graphs or maps. We do so also in daily communication when we use spatial language, terms such as *under, over, to the left of* or *in front of*, and when we give instructions. Spatial terms appear in children’s early vocabularies and continue to develop until late childhood or even early adolescence. Because spatial language develops over many years, some spatial terms are mastered very early, whereas others take longer to acquire. In the current set of studies, we tested how intellectually high-functioning children and adults on the autism spectrum use and understand these early- and late-acquired spatial terms in comparison to typically developing age-matched individuals. We found that children and adults on the autism spectrum experience difficulties with the use of some spatial terms (e.g. *near* and *far* or *out of* and *down off*) but not with others, which are acquired early (e.g. *in* and *on* or *over* and *under*). We also found that remembering spatial terms from short stories was more difficult for the individuals on the autism spectrum compared with typically developing individuals. These results reveal difficulties that can profoundly affect everyday communication of children and adults on the autism spectrum but also open new directions of research on language development in autism spectrum disorders.

## Introduction

Spatial skills are a core component of cognitive development and have a unique role in predicting later performance in a number of areas, including mathematics and science (Rimfeld et al., 2017; Verdine et al., 2017). Furthermore, spatial thinking is ubiquitous in everyday life and necessary for successful locomotion, wayfinding and tool use. Even though visuospatial abilities have been considered a strength in autism spectrum disorder (ASD; Mitchell & Ropar, 2004; Mottron et al., 2006; Stevenson & Gernsbacher, 2013), a growing number of studies point to difficulties in spatial tasks in ASD. Importantly, these difficulties were also observed in intellectually high-functioning individuals with ASD (here referred to as HFA), who score within normal ranges on standardized tests of cognitive and language abilities. There is evidence for selective impairments in HFA in spatial working memory (Lai et al., 2017; Wang et al., 2017), visual perspective taking (Pearson et al., 2013; Shield et al., 2016), binding objects to locations (Ring et al., 2015) and spatial navigation (Lind et al., 2013, 2014; Ring, Gaigg, de Condappa, et al., 2018; Smith, 2015). Crucially, acquisition of these skills goes hand in hand with linguistic development and involves the mastery of spatial language, that is, verbal descriptions of spatial relations such as *under, to the left of, north* or *towards*.

Spatial language is a unique domain of language, which is tightly yoked to non-verbal spatial abilities (Coventry & Garrod, 2004; Hayward & Tarr, 1995; Landau & Hoffman, 2005; Landau & Jackendoff, 1993) and builds on the pre-linguistic concepts already present from the first months of life (Casasola, 2018). The acquisition of spatial terms is strikingly consistent across languages, with *in*/*on, up*/*down, here*/*there* and *over*/*under* mastered first, followed by projective prepositions *front*/*behind* and *right*/*left*, which are not fully acquired before age 5 or 6 (Clark, 1973; Durkin, 1981; Harris, 1972; Johnston, 1988; Johnston & Slobin, 1979; Kuczaj & Maratsos, 1975; Landau & Hoffman, 2005). Mastery of geocentric terms (*north*/*south*) typically requires even more time (but see Shusterman & Li, 2016). Path terms for describing dynamic spatial relations (motion events) appear by age 2 (Brown, 1973; Choi & Bowerman, 1991), with goal paths (*into, onto*) encoded more often than source paths (*out of, down off*, see Lakusta & Landau, 2005, 2012; Papafragou, 2010). The well-organized and orderly development of spatial language allows for a clear distinction between the early- and late-emerging linguistic abilities. Importantly, even in the presence of a severe spatial impairment, as in Williams syndrome, the early-emerging spatial terms can be adequately mastered; however, late-emerging terms, such as projective prepositions, appear to be more vulnerable to spatial, or more generally, developmental deficits (Landau & Hoffman, 2005; Landau & Zukowski, 2003).

Surprisingly, spatial language abilities have never been thoroughly studied in ASD. Only a few studies, although with limited testing batteries and samples, reported evidence suggesting difficulties in spatial language in this population. In these studies, low-functioning children with ASD showed deficits in the comprehension and production of selected spatial and temporal prepositions (Churchill, 1972; Perkins et al., 2006; Ricks & Wing, 1975). Some preliminary observations have been also made about spatial language difficulties in individuals on the high end of the spectrum. In an early study by Ohta (1987), a subset of the sample constituted intellectually high-functioning children with ASD. In this study, one of the tasks tapped into the comprehension of spatial terms. Participants were verbally instructed to move certain items to new locations, for example, ‘put the button *on* the box’, or ‘put the button *next to* the box’. Results pointed to a significantly lower performance in this task in the ASD group compared with the control group, a difference that could not be solely explained by the general intelligence quotient (IQ). Observed errors included semantic violations of the prepositions, for example, putting the button *into* the box instead of *on* the box or *on top of* the box instead of *next to* the box. More recently, Vulchanova et al (2012, 2013) reported two case studies of individuals with HFA, who also performed surprisingly poorly on the spatial language task where they were asked to combine spatial prefixes (which in Slavic languages are semantically related to spatial prepositions) with the verbs. Still, many aspects of spatial language abilities have been largely ignored, for example, is spatial language affected uniformly in HFA or are difficulties limited to only certain types of spatial terms? Do these difficulties extend to comprehending and memorizing spatial descriptions, an ability essential when receiving verbal descriptions of locations or directions? Finally, are these difficulties related to changing perspectives in spatial language use, such as viewer-centred (e.g. ‘to my left’) versus environment-centred descriptions (e.g. ‘in front of the building’)?

Given that language deficits in the spatial domain can have profound consequences for education, as well as daily communication about objects’ locations, tool manipulation or navigation, the identification of such difficulties could lead to changes in the intervention targets in HFA. Moreover, the uneven cognitive and language profiles observed in ASD, with ‘peaks’ and ‘troughs’ within certain domains (Bernardino et al., 2012; Charman et al., 2011; Dawson et al., 2007; Lincoln et al., 1995; Mayes & Calhoun, 2003; Vulchanova et al., 2013), offer a means of addressing important theoretical questions. Specifically, can spatial language show an uneven breakdown, with some spatial language abilities being impaired while others are intact? Furthermore, are some components of spatial language more vulnerable in atypical linguistic or cognitive development? Finally, what type of developmental mechanism could account for this potential selective breakdown and could it explain the puzzle of uneven linguistic and cognitive development in ASD? Employing a novel spatial language battery, we present the first comprehensive test of spatial language abilities in HFA, attesting selective difficulties in the production of spatial terms and in the recall of spatial descriptions, consistent with an uneven cognitive profile.

## Methods

### Participants

Twenty-five intellectually high-functioning individuals with ASD (7 females, age range: 9–27, M = 17.9, standard deviation (SD) = 5.9) and 25 typically developing (TD) controls (11 females, age range: 9–27, M = 17.8, *SD* = 5.3), all native speakers of Norwegian, participated in the study (see Appendices 1 and 2 for the complete information about individual participants’ age and gender). The participants were recruited through the national and local branches of the Autism Society in Norway and local schools. Only individuals who had received a formal diagnosis of ASD or Asperger syndrome from an authorized psychologist in Norway (according to the *DSM*-4 criteria, and as such, automatically qualified for the diagnosis of Autism Spectrum Disorder under the 5th edition of the Diagnostic and Statistical Manual of Mental Disorders, *DSM*-5; American Psychiatric Association, 2013) were included in the HFA group. We included only individuals without intellectual disability (Full Scale IQ scores higher than 70, according to the *DSM*-5 cut-off point for intellectual disability; see Appendix 1 for the complete list of the individual Full Scale IQ scores in the HFA group).

**Figure 1.**
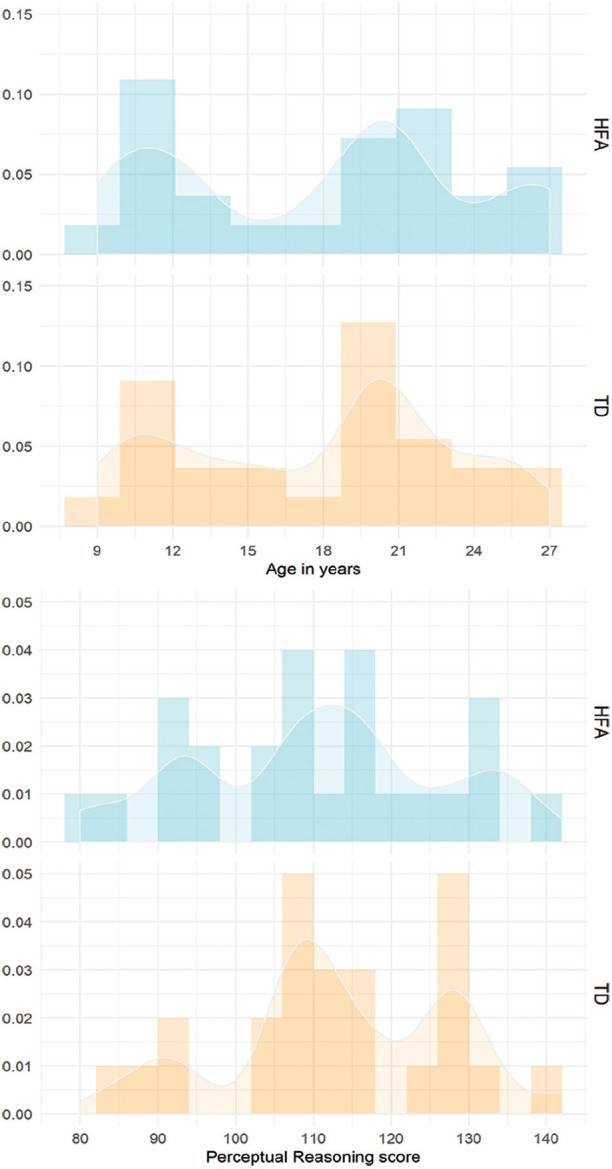
Distribution of age (upper panel) and Perceptual Reasoning scores (lower panel) in the HFA and TD groups. The y-axis represents density. HFA: high-functioning ASD; TD: typically developing.

Groups were matched on chronological age and Perceptual Reasoning (Wechsler IQ Scales; Wechsler, 2003, 2008; Norwegian standardization editions: Wechsler, 2009, 2011, respectively; see Table 1 and Figure 1). To compare the language abilities of the participants, we employed the overall Verbal Comprehension subscale (Wechsler IQ Scales). In addition, we assessed participants’ expressive language using the Speaking subscale (Sentence Combining and Multiple Meanings subtests) from the Test of Language Development–Intermediate: 4th Edition (TOLD-I:4; Hamill & Newcomer, 2008). We also obtained additional information about the ASD symptomatology in the HFA group and possible autism spectrum traits in the TD group using the autism-spectrum quotient (AQ; Baron-Cohen et al., 2001; Norwegian translation) and the Childhood Autism Spectrum Test (CAST; Scott et al., 2002; Norwegian translation) questionnaires (see Appendices 1 and 2 for the complete list of the individual AQ and CAST scores in the HFA and TD groups). The AQ and CAST questionnaires are brief instruments that allow measurement as to where any given individual lies on the continuum of autism spectrum traits (Baron-Cohen et al., 2001). In the present sample, the average proportions of the questionnaire scores differed significantly between the groups, with the HFA participants scoring reliably higher than controls (see Table 1).

**Table 1.** Descriptive characteristics of the HFA and TD groups.

Variable	Assessment	HFA (*N* = 25)	TD (*N* = 25)	*p*-value (independent samples t-tests)
		M (SD)	M (SD)	
Chronological age		17.9 (5.9) range: 9–27	17.8 (5.3) range: 9–27	*p* = 0.921
Gender (M/F)		18/7	14/11	
Perceptual Reasoning	Perceptual Reasoning Index WISC-IV or WAIS-IV	110.80 (16.286)	113.56 (14.417)	*p* = 0.529
Verbal Comprehension	Verbal Comprehension Index WISC-IV or WAIS-IV	106.00 (14.754)	112.36 (9.032)	*p* = 0.073
Expressive language	Speaking subscale TOLD-I:4	0.71 (0.12)	0.77 (0.11)	*p* = 0.06
Autistic traits/symptomatology	AQ/CAST Questionnaire	0.56 (0.16)	0.15 (0.12)	*p* < 0.001***

HFA: high-functioning ASD; TD: typically developing; WISC-IV: Wechsler Intelligence Scales for Children (4th Edition); WAIS-IV: Wechsler Adult Intelligence Scales (4th Edition); TOLD-I:4: Test of Language Development–Intermediate (4th Edition); AQ: autism-spectrum quotient; CAST: Childhood Asperger Syndrome Test; ASD: autism spectrum disorder; SD: standard deviation.

*** *p* ≤ .001.

### Materials

We adapted a battery of spatial language tests developed at the University of East Anglia (Markostamou & Coventry, 2020a, 2020b; Markostamou et al., 2015). The battery includes the Spatial Naming Test (SNT), the Spatial Verbal Memory (SVM) task and the Rotating Board Spatial Referencing (RBSR) task (the order corresponds to the order of administration).

**Figure 2.**
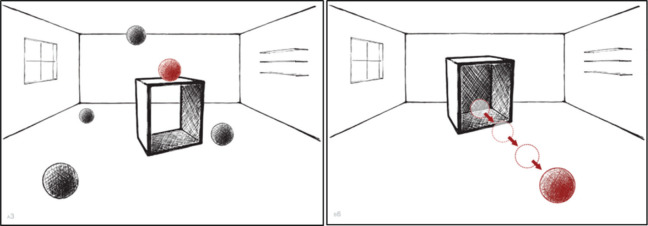
Example stimuli from Spatial Naming Test: part A (locative prepositions; left panel) and part B (directional/path prepositions; right panel).

The SNT is an analogue to the Boston Naming Test (Kaplan et al., 2001) and tests production of locative and directional/path prepositions. It consists of 30 pictures with simple geometrical shapes that represent different types of spatial relations (see Figure 2). The participant’s task was to name as accurately as possible the red ball’s position or its direction of movement in relation to the black cube, as distinguishable from the black balls’ locations. Part A (15 items) included relations denoted by locative prepositions: *in, on, to the right of, on the left of, beside, above, under, below, behind, in front of, far to the left of, near to the left of, between, among* and *in the middle*. Part B (15 items) included motion events, which target directional and path prepositions: *downwards, upwards, to the right, across, into, onto, towards the side, out of, away from, down of, around, over, under, through, and along.*

The SVM task consists of two short stories told from egocentric (route description) and allocentric (survey description) perspectives matched for number of words and propositions. The egocentric (route description) stories described the spatial locations of the landmarks from the perspective of an agent, for example, ‘When he saw the blue lake in front of him, he turned left’, whereas the allocentric (survey description) stories described the spatial locations of the landmarks from the overhead, bird’s-eye view, for example, ‘The City Hall is in the centre of the town’. The stories were translated into Norwegian and pre-recorded by a native Norwegian speaker (see Appendix 3 for the original and translated versions of the stories). The participant’s task was to listen to the story and subsequently verbally recall everything they remembered from the story (as close as possible to how it was told). Around 25 minutes after immediate recall, participants were asked to retell the stories one more time (delayed recall).

**Figure 3.**
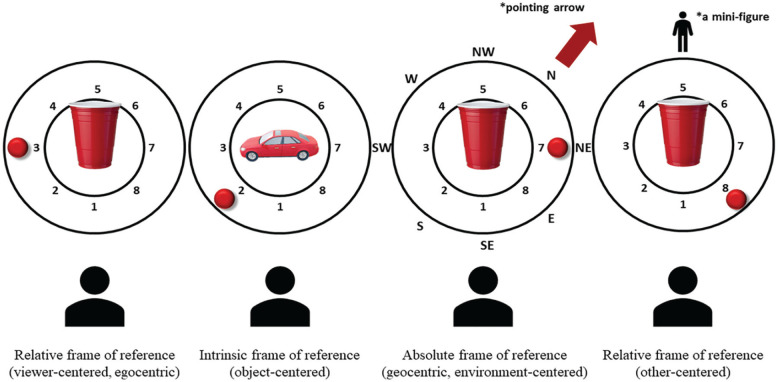
Rotating Board Spatial Referencing. Four task conditions, relative: viewer centred, intrinsic: object centred, absolute: environment centred and relative: other centred. Note that the boards, shown in plan view here, were presented horizontally (flat) in front of the participant. Numbers and letters that mark the locations in the picture were not visible to the participants.

The Rotating Board Spatial Referencing (RBSR) task tested comprehension of spatial terms from different spatial perspectives: intrinsic (object centred), absolute (geocentric, environment centred) and relative (egocentric: viewer centred and other person centred). Materials consisted of a rotating board with a red ball mounted on the outer rotating ring with the inner space in the middle reserved for the reference object (a cup or a toy car, depending on the condition; see Figure 3). The participant’s task was to judge the statements about the ball’s position as ‘true’ or ‘false’. More specifically, the experimenter on each trial (16 trials per condition) moved the ball to one of its pre-defined positions (see the locations numbered 1–8 in Figure 3) and read out loud a statement about the ball’s position, for example, ‘The ball is behind and to the right of the cup’. The participant responded ‘true’ or ‘false’ to every statement. In the relative condition, participants were judging whether the statements were true or false from their perspective (viewer centred) or from the perspective of the mini-figure on the other side of the board (other person centred). In the intrinsic condition, the participants were judging whether the statements were true or false from the perspective of an object with a natural front and back (a toy car). In the absolute condition, participants were judging as true or false statements such as ‘The ball is southeast of the cup’ with an arrow pointing to the north as the reference (see Figure 3).

### Procedure

The study was conducted in compliance with the Regional Committees for Medical and Health Research Ethics (REK) in Norway (reference number: 2015/1642; project title: ‘Spatial language and spatial cognition in Autism Spectrum Disorder’). Participants (older than 18 years) or their parents (for participants younger than 18 years) filled out and signed the consent form for voluntary participation in the study. Participant assent was also obtained for children under the age of 18. All participants and the parents were informed about participation requirements and study procedures, and detailed instructions were given before each task. The total amount of time required to complete the spatial language battery was about 30–40 minutes. Background measures were collected independently in a separate testing session (total time about 2.5 hours) and included the Wechsler Intelligence Scale (WISC-IV for participants under 16 years old and WAIS-IV for participants over 16 years old), Test of Language Development (TOLD-I:4) AQ (for participants over 16 years) and the CAST (the parents of participants younger than 16 years) questionnaires. Testing took place at The Norwegian University of Science and Technology and at the University of Oslo. At the end of the testing procedure, all participants were invited to choose a gift (from among board games, puzzles, bags, cinema or water park tickets) in compensation for their participation in the study.

## Results

### Spatial Naming Test

All collected answers from the SNT were rated by two independent raters (a professional linguist and a linguist in training; both native speakers of Norwegian) on a 5-rank scale with respect to the semantic content of the response, that is, how accurately it corresponded to the content of the picture (very accurate, accurate, acceptable, barely acceptable or not acceptable). To ensure non-biased rating, the answers were arranged in alphabetical order under every item and not assigned to any subject codes, so that the raters were blind to the age, gender or diagnostic classification of the participants. Rated answers were subsequently scored by the experimenter (full score = 1, if the answer was rated as very accurate or accurate, half score = 0.5, if the answer was rated as acceptable, and 0 score, if the answer was rated as not acceptable).

The raters showed full agreement in 58% of the rated items and disagreement on 4% of the rated items. For the rest of the items (38%), the raters were in partial agreement, that is, one rater indicated an item as accurate and the other one as acceptable. In the cases of partial agreement or disagreement, the highest rating decided about the score for the particular answer (i.e. if at least one rater indicated that the answer was accurate, it received a score of 1; similarly if at least one rater indicated that the answer was acceptable, it received a score of 0.5).

**Figure 4.**
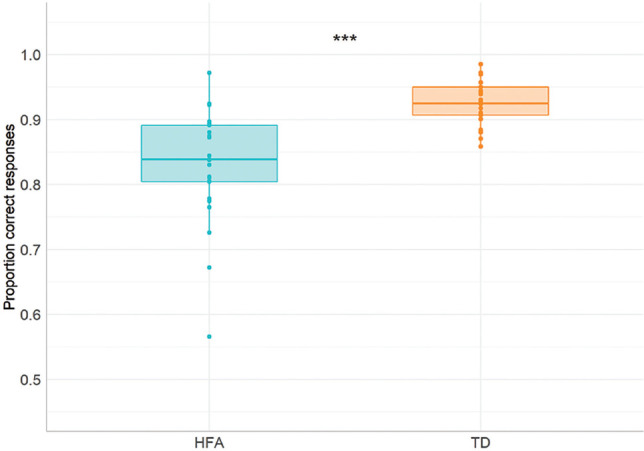
Proportion correctly named items in the Spatial Naming Test in the HFA and TD groups. HFA: high-functioning ASD; TD: typically developing. ***p ⩽ 0.001.

We first looked at the overall performance between the groups in the task. We ran a 2 × 2 analysis of variance (ANOVA) on the average test scores with Preposition Type (locative, directional/path) as a within-subject factor and Group (HFA and TD) as between-subject factor. We added Verbal Comprehension and Expressive language scores as a covariate in the analysis to control for possible group effects caused by differences in verbal abilities (see relatively low *p*-value in Verbal Comprehension and Expressive language comparisons, Table 1). The analysis revealed a significant main effect of Group, *F*(1,49) = 19.643, *p* < 0.001, $\eta_{p}^{2}$  = 0.309, with the HFA group scoring overall lower on the test compared with controls (see Figure 4). There was no main effect of Preposition Type, *F*(1,49) = 0.003, *p* = 0.96, $\eta_{p}^{2}$  < 0.001 or significant Preposition Type * Group interaction, *F*(1,49) = 0.012, *p* = 0.91, $\eta_{p}^{2}$ < 0.001.

Next, we looked at the accuracy scores on single-test items to investigate whether spatial language production was affected uniformly or selectively (see Appendix 4 for the complete summary of the comparisons). While on some items, both groups were at ceiling (e.g. *in, under, upwards*), the highest discrepancies between the groups’ scores were in proximal (*near*/*far*) and source path terms (*out of*/*down off*/*away from*). After collapsing these item scores into two categories, Mann–Whitney U tests confirmed that in comparison to controls, the HFA group made significantly more errors both in the Proximity, U = 113, *p* < 0.001, and Source Path category, U = 166, *p* = 0.003 (see Appendices 5 and 6 for a qualitative description of error types).

### Spatial Verbal Memory Task

Participants’ answers from both stories in the SVM task were recorded and later transcribed. Each story was divided into 25 items: 10 spatial items (e.g. ‘towards’, ‘in front of him’, ‘on his right’) and 15 non-spatial items (e.g. ‘started walking’, ‘the City Hall’, ‘the Museum’). Every correctly recalled item received 1 point. We calculated the proportions of correctly recalled items separately for spatial and non-spatial content of the stories and conducted full-factorial analyses on the proportions.

**Figure 5.**
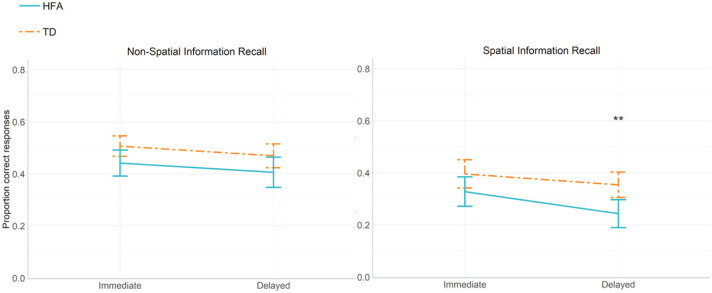
Proportion correctly recalled non-spatial and spatial items in the Spatial Verbal Memory task (immediate and delayed recall); error bars represent ±2 SEM. **p ⩽ 0.01.

We ran a 2 × 2 ×2 × 2 ANOVA, with Reference Frame (egocentric and allocentric), Recollection Time (immediate and delayed) and Item Type (spatial and non-spatial) as within-subject factors and Group (HFA and TD) as a between-subject factor on the proportions of correctly recalled items, with Verbal Comprehension and Expressive language as covariates. We observed a significant main effect of Group, *F*(1,49) = 4.193, *p* = 0.047, $\eta_{p}^{2}$ = 0.093, with the TD group scoring on average better on this task (M = 0.425, SE = 0.022) than the HFA group (M = 0.360, SE = 0.022). There was also a significant Item Type * Recollection Time * Group interaction, *F*(2,48) = 4.408, *p* = 0.042, $\eta_{p}^{2}$ = 0.097, displayed in Figure 5. Post hoc comparisons (with Bonferroni corrections) revealed a significant difference between the groups only in the delayed recall of spatial information condition, *t*(2,48) = –3.222, *p* = 0.008, with lower recall scores in the HFA group compared with the TD group. None of the other comparisons reached significance (see Figure 5).

Finally, in order to control for possible differences in working memory abilities between the TD and HFA groups, we compared the Working Memory subscale scores (Wechsler IQ test) between the groups and used the working memory scores as a predictor for performance of participants with HFA in the delayed recall of the SVM task. The analyses showed no significant differences in the working memory scores between the groups, *t* = –1.351, *p* = 0.183 and no significant effect of working memory scores on the delayed recall in the HFA group, *R*^2^ = 0.0259, *F*(1,24) = 1.2, *p* = 0.28.

### Rotating Board Spatial Referencing Task

Four participants (two HFA and two TD) withdrew before the completion of the last task, and therefore, the group statistics are different for the RBSR task (see Table 2).

**Table 2.** Descriptive characteristics of the HFA and TD groups: Rotating Board Spatial Referencing Task.

Variable	Assessment	HFA (*N* = 23)	TD (*N* = 23)	*p*-value (independent samples t-tests)
		M (SD)	M (SD)	
Chronological age		18.1 (6.1) range: 9–27	18.1 (5.2) range: 9–27	*p* = 0.979
Gender (M/F)		16/7	11/12	
Perceptual Reasoning	Perceptual Reasoning Index WISC-IV or WAIS-V	111.35 (16.535)	113.91 (13.804)	*p* = 0.571
Verbal comprehension	Verbal Comprehension Index WISC-IV or WAIS-V	108.57 (12.284)	113.61 (8.261)	*p* = 0.109
Expressive language	Speaking subscale TOLD–I;4	0.71 (0.12)	0.78 (0.11)	*p* = 0.03*
Autistic traits/symptomatology	AQ/CAST questionnaire	0.56 (0.16)	0.15 (0.12)	*p* < 0.001***

HFA: high-functioning ASD; TD: typically developing; WISC-IV: Wechsler Intelligence Scales for Children (4th Edition); WAIS-IV: Wechsler Adult Intelligence Scales (4th Edition); TOLD-I:4: Test of Language Development Intermediate (4th Edition); AQ: autism-spectrum quotient; CAST: Childhood Asperger Syndrome Test; ASD: autism spectrum disorder; SD: standard deviation.

* *p* ≤ .05; ***p ≤ .001.

**Figure 6.**
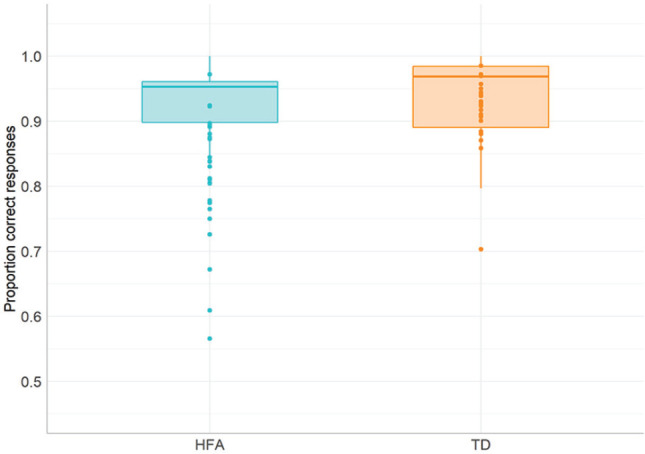
Average accuracy scores in HFA and TD groups in the Rotating Board Spatial Referencing task. HFA: high-functioning ASD; TD: typically developing.

We first compared the overall accuracy on the task and ran non-parametric tests for between subject comparisons, because of strongly skewed distributions of the scores (with skewness of −1.82, SE = 0.35, and kurtosis of 3.37, SE = 0.69). Mann–Whitney U test on the scores showed no reliable differences overall between the groups on the task (U = 197, *p* = 0.137; see Figure 6). In order to investigate whether individuals with HFA showed selective difficulties with only one and not all of the task conditions, we calculated the proportions of correct responses for each condition. Mann–Whitney U tests revealed a significant difference between the groups in the egocentric (viewer centred) condition (U = 357, *p* = 0.008), with the HFA group scoring significantly lower compared with the TD group (note, however, that HFA participants scored still around 90% in that condition, which indicates an overall successful performance in that condition despite observed group difference). None of the other comparisons reached significance.^1^

In order to identify what types of errors contributed to the group differences in the egocentric condition, we identified the items with the highest discrepancy in the number of errors between the groups. Items ‘in front of’, ‘in front of and to the left of’ and ‘behind’ showed the highest percentage of errors in the HFA group (13%, 13% and 9%, respectively), while TD participants’ scores were at ceiling. Qualitative analysis of the incorrect answers revealed that participants with HFA interpreted the direction within front/back axis differently compared with the TD group. Instead of using the mirror reflection of the axis, where Front is on the same side of the reference object as the viewer, a translation strategy was used, where Front is placed on the opposite side of the reference object.^2^ However, this strategy was not applied systematically throughout this condition.

### Performance predictors

**Figure 7.**
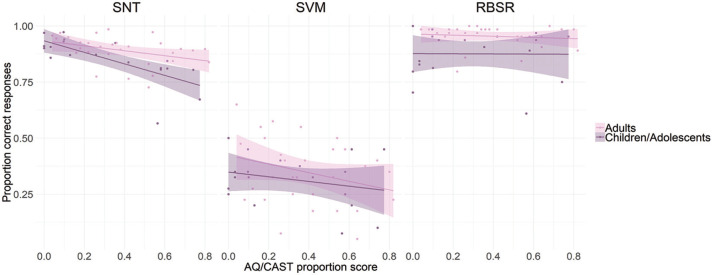
The relationship between task accuracy and proportion obtained score in the AQ/CAST questionnaire. AQ/CAST scores significantly predicted performance in the SNT and SVM task (left and middle panel) but not in the RBSR task (right panel). The relationship has been plotted separately for two age groups (for visualization purposes only): children/adolescents (9–17 years) and adults (18–27 years); shaded area represents ±2 SEM. SNT: Spatial Naming Test; SVM: Spatial Verbal Memory; RBSR: Rotating Board Spatial Referencing; AQ: autism-spectrum quotient; CAST: Childhood Asperger Syndrome Test.

Finally, we investigated whether the level of autism spectrum traits, as measured by the scores obtained on the AQ/CAST questionnaires, or participants’ age could account for the differences in task performance (see Figure 7). We ran multiple regression analyses with the average proportion of AQ/CAST scores and age as predictors, separately for the scores in the SNT, SVM and RBSR tasks. The results of the regression analyses indicated that the two predictors explained 40% of the variance in the SNT task (*R*^2^ = 0.396, *F*(2,46) = 15.1, *p* < 0.001), with both AQ/CAST score (*β* = –0.19, *t* = –4.96, *p* < 0.001) and age (*β* = 0.006, *t* = 3.22, *p* = 0.002) significantly predicting task performance.

In the SVM task (Spatial Delayed condition), the model accounted for 14% of the variance (*R*^2^ = 0.142, *F*(2,46) = 3.55, *p* = 0.037), where only AQ/CAST score (*β* = –0.20, *t* = –2.58, *p* = 0.013), but not age (*β* = 0.003, *t* = 1.10, *p* = 0.278), significantly predicted task performance.

In the RBSR task, the model explained 11% of the variance (*R*^2^ = 0.111, *F*(2,43) = 2.7, *p* < 0.079), where only age (*β* = 0.005, *t* = 2.3, *p* = 0.026), and not AQ/CAST score (*β* = –0.009, *t* = –0.173, *p* = 0.864), accounted for task performance. In sum, the level of autism spectrum traits predicted performance in the SNT and SVM task and age-predicted participants’ performance in the SNT and RBSR task.

## Discussion

The current study revealed selective difficulties in HFA in the spatial language domain. Specifically, individuals with HFA scored lower than controls on spatial language production and spatial description recall; however, these difficulties were not distributed uniformly but rather clustered in the areas of projective prepositions (*left/right, front/back*), source path terms (*out of/down off)*, proximal terms (*near/far*) and delayed recall of spatial content. These findings provide the first evidence for selective deficits in a broad range of spatial language abilities in ASD that are also positively associated with autism spectrum traits. Finally, contrary to previous evidence suggesting difficulties with perspective taking in ASD (Pearson et al., 2013; Shield et al., 2016), the HFA participants in the current study showed overall similar performance to controls on the RBSR task.

Our results provide new, clinically relevant insights into the characteristics of the linguistic profiles in HFA. The findings indicate selective deficits in the spatial language domain, which lead to an uneven profile that resembles the uneven profiles reported previously for other aspects of cognition and language in HFA. For example, despite their relatively large vocabularies, individuals with HFA have been shown to display selective deficits in lexical processing (e.g. the use of idiosyncratic meanings and the absence of a shape bias in word learning, see Tek et al., 2008; Tek & Naigles, 2017; Volden & Lord, 1991). Similarly, individuals with HFA were shown to master most aspects of grammar but nevertheless display subtle difficulties with some syntactic elements, such as double complement constructions or personal and reflexive pronouns (Eigsti et al., 2007; Janke & Perovic, 2015, 2017; Kjelgaard & Tager-Flusberg, 2001; Perovic et al., 2013a, 2013b). Such selective and nuanced deficits in language are often disregarded in clinical work and absent from intervention targets, as they seemingly do not have a significant effect on the everyday functioning of individuals with HFA. Even though these difficulties often do not pass the impairment threshold in standardized tests, they should be nevertheless addressed in both clinical work and basic research, as they can have a profound impact on the overall functioning of individuals with HFA (Eigsti & Schuh, 2017), and they are often one of the strongest predictors of outcomes even on the high end of the spectrum (Szatmari et al., 2003).

Beyond their obvious clinical significance, the current results also carry significant theoretical implications. First, they provide new evidence for a dissociative nature of spatial language, that is, a possibility for impairment in some subsystems (e.g. production of projective, proximal and source path terms), while other subsystems remain intact. Second, they can give new insights into possible mechanisms behind this selective breakdown. Here, a critical question is whether one developmental mechanism, such as a developmental delay (i.e. a later acquisition of certain spatial language abilities compared with the timing in typical development) or arrest (i.e. a failure to acquire certain spatial language abilities at any point in development) could account for all selective deficits in spatial language in HFA.

Many of the deficits we observed in our HFA population corresponded to late-emerging abilities in spatial language and show parallels with other developmental disorders, for example, problems with projective prepositions and source paths have been previously observed in both younger children and individuals with Williams syndrome (Durkin, 1981; Harris, 1972; Johnston, 1984; Landau & Hoffman, 2005; Landau & Lakusta, 2006; Landau & Zukowski, 2003). This can indicate a delay in the mastery of terms, which are acquired later in development and which appear more challenging in the type of mapping between the language and the visuospatial representations. In further support of the delay hypothesis, the present study showed that both AQ/CAST scores and age predicted participants’ production of spatial terms. This suggests that irrespective of the intensity of autism spectrum traits within our population, performance could still improve with age. Accordingly, as relational memory (e.g. binding objects and locations in memory) shows a protracted development but single-item memory develops early (Ngo et al., 2017), the difficulties we observed in the recall of spatial descriptions could also result from a developmental delay or arrest. Interestingly, we found significant group differences only in the delayed, and not immediate, recall of spatial content. This difference was not accounted for by general working memory abilities in the HFA group. One possible explanation for this finding is that as verbal and visuospatial components are involved in memorizing spatial descriptions, but to different degrees (Brunyé & Taylor, 2008; De Beni et al., 2005), the participants with HFA might have engaged verbal working memory in the immediate recall (see Williams et al., 2005), while only delayed recall relied on relational memory. As a result, only delayed recall of spatial content posed difficulties in the HFA group as it relied on a cognitive ability that is acquired later in development. This would indicate that, even though individuals with HFA might not differ in their overall working memory abilities from the TD individuals, they might show difficulties in binding separate items in memory from language, an ability that is foundational to encoding and retrieving relational information from spatial language.

However, some of the observed deficits, such as the omissions of proximal terms and the lack of group differences in the RBSR, are difficult to reconcile with the developmental delay hypothesis. Proximal terms (*near*/*far*) are acquired relatively early in development and often even spontaneously chosen over projective prepositions by Williams syndrome individuals or typical children (Landau & Hoffman, 2005). Thus, observed omissions of proximal terms in the HFA group might result from a different mechanism, for example, a failure to attend to the distance in the spatial configuration between the located and the reference object (or interpreting it as less salient). Alternatively, the omissions might also point to a more specific problem with proximity in ASD (cf. the use of *here* and *this* for distal locations in ASD; Hobson et al., 2010). Given that proximal terms are imprecise (not defining the exact distance from the reference object), highly context-dependent and subjectively evaluated (Durkin, 1981), they might pose a particular challenge in ASD (Jolliffe & Baron-Cohen, 1999; Lopez & Leekam, 2003; Vermeulen, 2015). Another possibility is that, in the current study, the proximity items required more advanced use of proximal terms. Indeed, it has been previously shown that the use of *near* and *far* changes over time and reaches adult-like level only later in development, showing a surprisingly protracted learning trajectory (Durkin, 1981).

Different mechanisms could also account for the lack of group differences in the RBSR task in the present study. One possibility is that the HFA group compensated by applying an inversion strategy (i.e. inverting the left-right and front-back axis), instead of rotating their mental position – a type of strategy observed in children in mental rotation tasks, which facilitates performance (see Vander Heyden et al., 2017). In this way, participants could arrive at the normalized response by applying alternative strategies, a mechanism previously observed in HFA with ‘optimal outcomes’ (e.g. Eigsti et al., 2016). Another possibility is that, as language codes space in a coarse manner as opposed to detailed perceptual coding (see Jackendoff, 1983; Landau & Jackendoff, 1993), the comprehension of spatial terms can, in fact, be easier than fine-grained visual comparisons in different spatial perspectives. This interpretation could explain the differences between the current study, which tested linguistic performance in a perspective taking task, and other studies that tested non-linguistic perspective taking in HFA (Pearson et al., 2013; Ring, Gaigg, Altgassen, et al., 2018; Shield et al., 2016). Since the current results do not provide sufficient evidence to resolve between these possibilities, future research should use more fine-grained measures, which tap into the online processes involved in the task.

In sum, the present findings not only provide novel evidence for spatial language difficulties in individuals on the autism spectrum but also indicate a dissociative rather than uniform nature of these difficulties and point to several possible (but not mutually exclusive) mechanisms underlying this selective breakdown. That is, some of the observed deficits could be accounted for by a developmental delay hypothesis, while others most probably result from different mechanisms, such as impairments in integrating linguistic and non-linguistic information or deficits in spatial cognition.

These findings suggest intriguing questions for further research. Specifically, do some of the observed deficits in spatial language in HFA reflect an underlying deficit in spatial cognition or arise on the intersection of language and cognition? Furthermore, what regulates the integration of linguistic and non-linguistic spatial information and which aspects of this process (if any) are affected in HFA? Finally, to what extent are some of observed deficits, such as difficulties with proximity terms, specific to ASD? Although further research is necessary, the current study provides the first evidence for a range of selective difficulties in spatial language in HFA, revealing the dissociative nature of the spatial language domain as well as yielding important insights for future clinical work.

## Appendix 1

**Table:** The full list of individual HFA participant’s age, gender as well as the IQ and AQ/CAST scores.

Participant	Group	Gender	Age	IQ full scale	AQ/CAST score
101	HFA	Male	13	77	18
102	HFA	Male	12	123	19
103	HFA	Male	12	94	8
104	HFA	Male	10	120	11
105	HFA	Male	10	102	23
106	HFA	Male	9	119	13
107	HFA	Female	13	91	24
108	HFA	Male	10	95	17.5
109	HFA	Male	11	88	18
110	HFA	Male	16	109	19
111	HFA	Female	18	109	37
112	HFA	Male	21	104	26
113	HFA	Male	27	101	32
114	HFA	Male	19	92	21
115	HFA	Female	21	88	13
116	HFA	Female	25	100	40
117	HFA	Female	20	102	34
118	HFA	Male	20	102	16
119	HFA	Male	21	98	19
120	HFA	Female	19	74	26
121	HFA	Female	27	110	41
122	HFA	Male	21	116	29
123	HFA	Male	22	122	32
124	HFA	Male	25	106	27
125	HFA	Male	27	116	27

HFA: high-functioning ASD; IQ: intelligence quotient; AQ: autism-spectrum quotient; CAST: Childhood Asperger Syndrome Test; ASD: autism spectrum disorder.

## Appendix 2

**Table:** The full list of individual TD participant’s age and gender as well as the IQ and AQ/CAST scores.

Subject	Group	Gender	Age	IQ full scale	AQ/CAST score
201	TD	Male	9	108	0
202	TD	Male	12	112	1
203	TD	Female	11	110	3
204	TD	Male	13	113	3
205	TD	Female	15	109	0
206	TD	Male	11	91	0
207	TD	Female	10	104	4
208	TD	Male	16	108	5
209	TD	Male	14	112	0
210	TD	Female	10	100	1
211	TD	Female	17	108	17
212	TD	Male	21	120	18
213	TD	Female	20	108	11
214	TD	Male	20	95	3
215	TD	Male	20	95	21
216	TD	Male	24	112	16
217	TD	Female	23	114	13
218	TD	Female	20	124	9
219	TD	Female	26	124	11
220	TD	Male	25	112	9
221	TD	Female	20	116	8
222	TD	Female	20	108	2
223	TD	Female	20	103	4
224	TD	Male	26	126	14
225	TD	Male	22	99	6

TD: typically developing; IQ: intelligence quotient; AQ: autism-spectrum quotient; CAST: Childhood Asperger Syndrome Test.

## Appendix 3

### Spatial Verbal Memory task materials: egocentric and allocentric stories (spatial information in bold)

#### Egocentric story: original

Alex was **on** the main path **at** the Great Mountain, and started walking **towards** the peak. When he saw the blue lake **in front of** him, he turned **left**. He kept the lake **on his right**, until he passed **under** a large oak tree. He then crossed **over** a wooden bridge, leaving the lake **behind** him. He continued walking **straight on** and after a while he reached the peak.

#### Egocentric story: Norwegian translation

Alex befant seg **på** hovedstien **til** det store fjellet og begynte å gå **opp mot** toppen. Da han fikk øye på det blå vannet **foran** seg tok han av **mot venstre.** Han hadde vannet **på sin høyre side** til han passerte **under** et stor eiketre. Idet han krysset **over** ei trebru, hadde han vannet **bak** seg. Han fortsatte videre **rett fram** og etter en stund nådde han fram til toppen.

#### Allocentric story: original

The City Hall is **in the centre of** the town. **Around** the City Hall are a number of buildings. The Library is situated **in front of** the Church and **to the right of** the City Hall. The Market is just **behind** the City Hall, **next to** the Museum. The Gardens are **nearby**, located **to the left of** the City Hall. **On** the main avenue, which runs **along** the City Hall, there are many pubs and restaurants.

#### Allocentric story: Norwegian translation

Rådhuset ligger **midt i** byen. **Rundt** rådhuset er det flere bygninger. Biblioteket ligger **foran** kirken og **til høyre for** rådhuset. Torget er like **bak** rådhuset **ved siden av** museet. Parken er like **i nærheten**, og ligger **til venstre for** rådhuset. **I** hovedgata, som går **langs** med rådhuset, er det mange puber og restauranter.

## Appendix 4

**Table:** Proportions correct responses in the Spatial Naming Task per single item.

Item	Target preposition	Group	N	Mean	SD	SE
A1	i; inni [in]	HFA	25	0.960	0.138	0.0277
		TD	25	1.000	0.000	0.0000
A2	til høyre for [to the right of]	HFA	25	1.000	0.000	0.0000
		TD	25	1.000	0.000	0.0000
A3	på; oppå [on]	HFA	25	1.000	0.000	0.0000
		TD	25	1.000	0.000	0.0000
A4	over [above; over]	HFA	25	0.840	0.374	0.0748
		TD	25	0.980	0.100	0.0200
A5	bak [behind]	HFA	25	0.880	0.332	0.0663
		TD	25	0.960	0.200	0.0400
A6	under [under]	HFA	25	1.000	0.000	0.0000
		TD	25	1.000	0.000	0.0000
A7	under; langt under [below]	HFA	25	0.920	0.277	0.0554
		TD	25	0.980	0.100	0.0200
A8	foran [in front of]	HFA	25	0.940	0.220	0.0440
		TD	25	1.000	0.000	0.0000
A9	langt **til venstre for** [far **to the left of]**	HFA	20	0.900	0.308	0.0688
		TD	25	0.880	0.332	0.0663
A9	**langt** til venstre for [**far** to the left of]	HFA	23	0.804	0.292	0.0608
		TD	24	0.979	0.102	0.0208
A10	nær **til venstre for** [near **to the left of]**	HFA	22	0.841	0.358	0.0764
		TD	23	1.000	0.000	0.0000
A10	**nær** til venstre for [**near** to the left of]	HFA	21	0.810	0.370	0.0807
		TD	22	1.000	0.000	0.0000
A11	inntil **den venstre siden av** [next to **the left side of**]	HFA	19	0.842	0.375	0.0859
		TD	20	1.000	0.000	0.0000
A11	**inntil** den venstre siden av [**next to** the left side of]	HFA	23	0.848	0.279	0.0583
		TD	25	0.920	0.187	0.0374
A12	mellom [between]	HFA	25	0.960	0.200	0.0400
		TD	25	1.000	0.000	0.0000
A13	blant [among]	HFA	25	0.300	0.323	0.0645
		TD	25	0.400	0.382	0.0764
A14	i midten av [in the middle of]	HFA	25	0.820	0.284	0.0569
		TD	25	0.920	0.187	0.0374
A15	**foran**; på motsatt side av	HFA	23	0.609	0.476	0.0992
	[**in front of**; on the opposite side]	TD	22	0.795	0.367	0.0783
	foran; **på motsatt side av**	HFA	17	0.529	0.329	0.0799
	[in front of; **on the opposite side**]	TD	23	0.826	0.324	0.0675
B1	nedover [downwards]	HFA	25	0.960	0.200	0.0400
		TD	25	1.000	0.000	0.0000
B2	oppover [upwards]	HFA	25	1.000	0.000	0.0000
		TD	25	1.000	0.000	0.0000
B3	mot høyre [to the right of]	HFA	25	0.960	0.138	0.0277
		TD	25	1.000	0.000	0.0000
B4	tvers over (fra venstre mot høyre)	HFA	25	0.880	0.299	0.0597
	(across (from the left to the right of))	TD	25	0.980	0.100	0.0200
B5	inn i [into]	HFA	25	0.920	0.277	0.0554
		TD	25	0.980	0.100	0.0200
B6	ut av; ut fra [out of]	HFA	25	0.800	0.382	0.0764
		TD	25	0.960	0.200	0.0400
B7	bort fra; vekk fra [away from]	HFA	25	0.460	0.455	0.0909
		TD	25	0.740	0.357	0.0714
B8	rundt [around]	HFA	25	1.000	0.000	0.0000
		TD	25	0.960	0.200	0.0400
B9	over [over]	HFA	25	1.000	0.000	0.0000
		TD	25	1.000	0.000	0.0000
B10	under [under]	HFA	25	0.920	0.277	0.0554
		TD	25	0.960	0.200	0.0400
B11	gjennom [through]	HFA	25	0.960	0.200	0.0400
		TD	25	0.960	0.200	0.0400
B12	opp på [onto]	HFA	25	0.760	0.411	0.0823
		TD	25	0.920	0.236	0.0473
B13	ned av; ned fra [down off]	HFA	25	0.760	0.293	0.0586
		TD	25	0.920	0.187	0.0374
B14	langs (til venstre foran) (along (to the left in front of))	HFA	25	0.600	0.433	0.0866
		TD	25	0.880	0.261	0.0523
B15	mot siden av; til sides for [towards the side of]	HFA	25	0.440	0.441	0.0881
		TD	25	0.660	0.426	0.0852

HFA: high-functioning ASD; TD: typically developing; ASD: autism spectrum disorder.

Note that some of the items were scored twice when it was possible to name two distinct components of a spatial relationship in the picture, for example, ‘far to the left side of’, with a proximity component ‘far’ and axial horizontal component ‘left side of’ (see e.g. item A9). In such cases, participants would get 1 point for correctly naming the proximity and 1 point for correctly identifying direction within horizontal axis. We observed highest differences in the scores in proximity components in items A9 and A10 (‘**far** to the left of’, ‘**near** to the left of’), axial horizontal component in item A10 (‘near **to the left of**’), and item A15 (‘on the opposite side of’). In addition, HFA group scored lower in items B6 (‘out of’), B7 (‘away from’) and B13 (‘down off’), representing bounded FROM path terms (see Jackendoff, 1983) or source paths, and item B14 (‘along’).

## Appendix 5

**Table:** Types of answers to the proximity items that were not rated as accurate or very accurate.

Answers to proximity items (not rated as accurate or very accurate)	HFA (*N* = 25)	TD (*N* = 25)
	Proportion answers	
1. Omission of proximity term *Ballen er* ***til venstre for*** *boksen* Ball-the is **to the left of** box-the (instead of «*nær til venstre for*», «near to the left»)	0.36	0.16
2. Semantic violation *Ballen er* ***langt unna*** *boksen* Ball-the is **far from** box-the (instead of «*nær*», «near»)	0.08	0
3. Not in relation to the reference object *Ballen er til venstre* ***nærmere hit*** Ball-the is to the left **closer to here** (insead of «*langt fra boksen*», «far from the cube»)	0.08	0
4. Alternative term *Ballen er* ***først til venstre*** Ball-the is **first to the left** (instead of «*nærmest*», «nearest»)	0.16	0.04
5. Perspective error *Ballen er* ***langt fram*** *til venstre* Ball-the is **far in the front** to the left (instead of «*langt til venstre*», «far to the left»)	0.16	0.04
6. Not specified description *Ballen er* ***nærmest til venstre*** Ball-the is **nearest to the left** (instead of «*inntil*», «touching»/«in contact»)	0.28	0.16

HFA: high-functioning ASD; TD: typically developing; ASD: autism spectrum disorder.

## Appendix 6

**Table:** Types of answers to the source path items that were not rated as accurate or very accurate.

Answers to source paths items (not rated as accurate or very accurate)	HFA (*N* = 25)	TD (*N* = 25)
	Proportion answers	
1. Omission of direction *Ballen beveger seg* ***fra*** *boksen* Ball-the is moving **from** box-the	0.32	0.24
2. Omission of source *Ballen beveger seg* ***ned*** Ball-the is moving **down**	0.16	0
3. Partial semantic violation *Ballen beveger seg* ***fra*** *boksen* Ball-the is moving **from** box-the (insead of «*ut fra*», «out of»)	0.16	0.08
4. Semantic violation *Ballen beveger seg* ***ut av*** *boksen* Ball-the is moving **out of** box-the (instead of «*bort fra*», «away from»)	0.32	0.12
5. Locative preposition *(. . .)* ***ved siden av*** *boksen* (. . .) **on the side of** box-the (instead of «*bort fra*», «away from»)	0.12	0.00
6. Direction error *Ballen beveger seg* ***til venstre*** Ball-the is moving **to the left** (instead of «*ned*», «down»)	0.08	0.00
7. Perspective error *Ballen beveger seg* ***til høyre*** Ball-the is moving **to the right** (instead of «*foran*», «in the front»)	0.16	0.00
8. Descriptive answers *Ballen starter oppå og går ned foran til høyre* Ball-the starts on top and goes down in front of to the right	0.16	0.12

HFA: high-functioning ASD; TD: typically developing; ASD: autism spectrum disorder.

## Notes

1. Despite the lack of significant differences in the distribution of male and female participants across the groups in the current study (SNT and SVM tasks: χ^2^ = 2.12, *p* = 0.145; RBSR task: χ^2^ = 2.24, *p* = 0.134), we also re-ran the analyses for all three tasks in order to see whether gender distribution could affect the results, as performance in spatial task can differ between males and females (Coluccia & Louse, 2004; Voyer et al., 1995, 2007). The analyses showed no significant effects of gender in any of the tasks.

2. The translation strategy is a common error observed early in development in young children (Clark, 1980; Cox, 1981; Harris & Strommen, 1972) and also utilized in certain languages, for example, in Hausa (Hill, 1975, 1982; Levinson, 2003). Thus, inversions of front and back terms are not an atypical behaviour but rather an error that can stem from a different interpretation of the layout or the spatial arrangement of the items in view or from a mild delay in the mastery of these terms (note that overall, the inversions were not applied systematically).
